# Recurrence and Survival Following Cytoreductive Surgery and Hyperthermic Intraperitoneal Chemotherapy for Synchronous and Metachronous Peritoneal Metastases of Colorectal Origin

**DOI:** 10.3390/cancers16030631

**Published:** 2024-02-01

**Authors:** Mette Fugleberg Nielsen, Sissel Ravn, Mette Møller Sørensen, Jonas Amstrup Funder, Lene Hjerrild Iversen

**Affiliations:** 1 Department of Surgery, Aarhus University Hospital, 8200 Aarhus N, Denmark; mefnel@rm.dk (M.F.N.); mette.moller.sorensen@rsyd.dk (M.M.S.); funder@clin.au.dk (J.A.F.); 2 Department of Clinical Medicine, Aarhus University, 8200 Aarhus N, Denmark

**Keywords:** recurrence, prognosis, colorectal cancer, cytoreductive surgery, hyperthermic intraperitoneal chemotherapy, synchronous peritoneal metastases, metachronous peritoneal metastases

## Abstract

**Simple Summary:**

Cytoreductive surgery (CRS) with hyperthermic intraperitoneal chemotherapy (HIPEC) has notably improved the 5-year survival in colorectal cancer (CRC) patients with peritoneal metastases (PM). This study investigated recurrence patterns and survival outcomes in patients with synchronous versus metachronous PM following complete CRS+HIPEC. Among 310 patients studied from June 2006 to December 2020, the recurrence rate was 79.7%, and sites were most frequently isolated peritoneal and multifocal recurrence. Recurrence locations did not differ between synchronous and metachronous PM groups. Notably, patients with metachronous PM had a shorter disease-free survival compared to synchronous PM (9.4 vs. 12.5 months, *p* = 0.01); however, the overall survival was similar. Despite frequent recurrence, especially at extraperitoneal sites, long-term survival was achievable after CRS+HIPEC in CRC patients with PM.

**Abstract:**

Cytoreductive surgery (CRS) with hyperthermic intraperitoneal chemotherapy (HIPEC) has improved the 5-year survival for colorectal cancer (CRC) patients with peritoneal metastases (PM). Little is known about recurrence patterns and recurrence rates between synchronous (S) and metachronous (M) PM following CRS+HIPEC. We aimed to describe the recurrence patterns, overall survival (OS) and disease-free survival (DFS) in S-PM and M-PM patients after complete CRS+HIPEC. From June 2006 to December 2020, a prospective cohort study included 310 CRC patients, where 181 patients had S-PM (58.4%) and 129 patients had M-PM (41.6%). After a median 10.3-month follow-up, 247/310 (79.7%) patients experienced recurrence, and recurrence sites included isolated peritoneal (32.4%), multifocal (peritoneal and liver and/or lung(s)) (22.7%), isolated liver (17.8%), isolated lung (10.5%) and other (16.6%) sites. Recurrence patterns did not differ between S-PM and M-PM. M-PM patients had an impaired DFS compared to S-PM patients (9.4 months (95% CI: 7.3–12.1) vs. 12.5 months (95% CI: 11.2–13.9), *p* = 0.01). The median OS was similar for S-PM and M-PM (38.4 months (95% CI: 31.2–46.8) vs. 40.8 months (95% CI: 28.8–46.8), *p* = 0.86). Despite frequent recurrence at extraperitoneal locations, long-term survival was achievable after CRS+HIPEC in CRC patients with PM. The recurrence patterns and OS did not differ between groups, yet M-PM patients had a shorter DFS.

## 1. Introduction

Colorectal cancer (CRC) is one of the most common types of cancer in Denmark with more than 4000 individuals affected each year [1]. Survival depends on the disease stage at the time of diagnosis, yielding a 3-year survival rate of 95% for patients with stage I disease, which declines to 23% in patients with known stage IV disease [2]. At the time of CRC diagnosis, 5–7% of patients present with synchronous peritoneal metastases (S-PM) [3,4,5], whereas 2–5.5% of patients develop metachronous peritoneal metastases (M-PM) after curatively intended resection [3,4,6]. The presence of PM has previously been considered as end-stage disease with no possible curative treatment, and the median overall survival is reported to be a few months despite amendment to best supportive care [7].

During the past decades, cytoreductive surgery (CRS) combined with hyperthermic intraperitoneal chemotherapy (HIPEC) has been established as a curatively intended treatment offered to a selected group of patients with PM of CRC origin [8]. In a few randomized clinical trials and several cohort studies, patients undergoing CRS+HIPEC repeatedly demonstrate 5-year survival rates of 33–51% [8,9,10,11,12]. However, the majority of CRC patients suffer from recurrence following complete CRS+HIPEC, with reported recurrence rates of app. 70% during 25 months follow-up [13]. The most compelling prognostic factors following CRS+HIPEC are the extent of peritoneal disease, which can be estimated using the Peritoneal Cancer Index (PCI) [14], and the completeness of the performed cytoreduction [15]. These prognostic factors are determined pre-, intra- and postoperatively. It is of interest to investigate potential prognostic factors that can be determined preoperatively and help guide clinical decision making. The debut (synchronous or metachronous) of PM may cause a different prognosis following CRS+HIPEC due to a different tumor biology and an inadequate initial treatment. It remains unclear and inconsistent what the prognostic implication of S-PM and M-PM might be [7,13,14,15,16,17,18]. Therefore, the current study aimed to describe the recurrence patterns, disease-free survival (DFS) and overall survival (OS) in patients with S- and M-PM following complete CRS+HIPEC in a national Danish cohort.

## 2. Materials and Methods

### 2.1. Design and Setting

This study was carried out as a national observational cohort study including patients undergoing CRS+HIPEC at a single national center (Aarhus University Hospital (AUH)) in the Central Denmark Region, Denmark. The Danish population is approximately 5.8 million people, of which approximately 1.2 million live in the Central Denmark Region. The Danish health care system, with five individual regions, provides tax-supported free health care services. CRS+HIPEC has been performed since June 2006 at the center, treating nationally referred PM patients.

### 2.2. Patients

In the period from June 2006 to December 2020, we included all patients who underwent CRS+HIPEC due to PM from a CRC origin. CRS+HIPEC was performed if no visible residual disease or residual tumor nodules ≤2.5 mm could be achieved, referred to as complete cytoreduction. Contraindications for CRS+HIPEC were a physiological age >75 years; an American Society Anesthesiologists (ASA) score ≥4; a WHO Performance score ≥2; extraperitoneal disease (except ≤3 liver metastases each with <3 cm eligible for radiofrequency ablation with curative intent or <2 lung metastases eligible for curative treatment); a Peritoneal Cancer Index (PCI) [19] score ≥17 (and ≥12 in case of curative liver metastases); and PM involving the caput pancreatis or causing biliary obstruction. The Dutch 7 Region Count Score [20] was used to assess the extent of peritoneal disease in 2006–2014, instead of the PCI score, and CRS+HIPEC was not offered if ≥6 regions were involved. Before 2016, patients with extraperitoneal metastatic disease were not offered CRS+HIPEC [21]. In a few cases (n = 8), patients underwent CRS+HIPEC despite the fact that the extent of PM exceeded the recommendations due to the assessment of the surgeons.

Preoperatively, patients underwent a positron emission tomography contrast-enhanced computer tomography (CT) of the chest, abdomen and pelvis and a colonoscopy if not performed within the previous 6 months. All patients were discussed in a multidisciplinary team conference with the participation of at least one CRS surgeon. In the case of uncertainty of the extent of PM, a diagnostic laparoscopy was performed.

### 2.3. Cytoreductive Surgery and Hyperthermic Intraperitoneal Chemotherapy

Initially, a laparotomy was performed with an evaluation of the extent of peritoneal spread assessed by the PCI score. The CRS procedure included the removal of tumor deposits by stripping the peritoneal surfaces, as described by Sugarbaker et al. [22]. Non-essential organs were removed if visceral peritoneal stripping was impossible. Liver surface involvement was managed via electrocoagulation or hepatic capsulectomy. Routine resections included the greater omentum, the umbilicus, the ligament teres hepatis and ovaries. After CRS, the peritoneal cavity was filled with peritoneal dialysis solution; from 2006–November 2016, high-dose mitomycin C 35 mg/m^2^ with triple dosing was used, with perfusion for 90 min at 41.0–42.5 °C, and from December 2016–2020, bidirectional intraperitoneal and intravenous treatment was given [23] using Oxaliplatin 260 mg/m^2^ with a perfusion time of 30 min and 5FU 500 mg/m^2^ Leucovorin 20 mg/m^2^, respectively. Mitomycin C was used in case of previous adverse reactions to Oxaliplatin [24]. After CRS+HIPEC, all patients were offered systemic adjuvant chemotherapy for 3–6 months unless the patient had received systemic chemotherapy for 6 months in a neoadjuvant setting. The choice of chemotherapy followed the existing national oncology guidelines at the time of surgery [25].

### 2.4. Follow-Up

All patients were offered consecutive follow-ups at 3, 6, 12, 18 and 24 months, as well as 3, 4 and 5 years after the surgery. Each follow-up was preceded with a contrast-enhanced positron emission tomography CT of the chest, abdomen and pelvis. Patients with recurrence were discussed at a multidisciplinary team conference regarding whether treatment with curative intent was possible or otherwise referred to their local hospital for palliation. A priori, patients received the results of the preceding imaging, but some patients had their follow-up at local oncological departments.

### 2.5. Data Collection and Variables

Patient data were extracted from a prospectively maintained database. In case of missing data, the information was retrieved from the electronic health record. The extracted clinical variables were the patient’s age, sex, ASA scores, primary tumor localization, previous cancer-related surgery, T- and N-category of primary CRC, presence of metastatic disease prior to CRS+HIPEC, preoperative chemotherapy, time from primary cancer resection to CRS+HIPEC, PCI score, chemotherapeutic agent used for HIPEC, and postoperative complication rate (any grade).

S-PM was defined as PM-CRC diagnosed at the time of the diagnosis of the primary tumor, during staging, at the time of the resection of the primary tumor or as an incidental finding at histopathological examination of the resected tumor specimen. PM-CRC diagnosed any time during follow-up was defined as M-PM. Peritoneal recurrence is defined as any metastasis within the peritoneal cavity and detected via imaging or clinically perioperatively. A histological confirmation was performed in case of inconclusive imaging and clinical treatment consequences.

Follow-up data (recurrence) were collected from the local database and/or from the electronic health record according to each follow-up visit. For patients who continued their follow-up outside the Central Denmark Region, we had only access to survival data and the national pathology data (all histopathological examinations). In such cases, a recurrence was only registered if a histopathological examination was performed.

The vital status (alive/dead) was obtained from the electronic health record.

### 2.6. Study Endpoints

The primary endpoint was a description of the recurrence pattern along with the disease-free survival (DFS) stratified by S-PM and M-PM.

The pattern of recurrence was defined as location-specific recurrence and was classified according to the anatomical site into “peritoneum”, “liver”, “lungs”, “multifocal (peritoneum and liver and/or lung(s))” and “other (abdominal wall, uterus, spleen, bones, cerebrum, adrenal gland)”. The site of recurrence was defined as the location of the first detected recurrence of disease.

The secondary endpoint was the overall survival (OS) stratified by S-PM and M-PM.

### 2.7. Statistical Analysis

Categorical variables are presented as numbers and percentages, while continuous variables are presented as the median with an interquartile range. Equality between groups (S-PM and M-PM) was tested using a chi-squared test for categorical variables and a Mann–Whitney U test for continuous variables.

The pattern of recurrence was presented as the proportion of patients with S-PM and M-PM, respectively, developing location-specific recurrence within the total follow-up period. The follow-up time was calculated from the date of CRS+HIPEC to recurrence, death or end of follow-up (10.06.2022). The DFS was defined as the time between CRS+HIPEC and the diagnosis of the first recurrence or date of the last follow-up in the Central Denmark Region. In case the patient was followed outside of the Central Denmark Region, patients were censored at the last date of follow-up in the Central Denmark Region, unless a histopathological biopsy verified recurrence. Patients were censored in case of death before recurrence or end of follow-up. Vital status was available for all patients.

The DFS and OS were determined for patients with S-PM and M-PM using the Kaplan–Meier method, and patients were monitored from the date of CRS+HIPEC until recurrence (DFS)/death (OS) or the end of the follow-up.

We performed three subanalyses of the DFS and OS: one where patients with extraperitoneal metastatic disease prior to CRS+HIPEC were excluded from the analysis and another where patients were stratified according to their receipt of neoadjuvant chemotherapy (yes/no) and finally a subanalysis of the DFS and OS stratified by pathology of CRS (mucinous adenocarcinoma vs. adenocarcinoma).

The equality of means in the DFS and OS was estimated using a log-rank test. All analyses were performed with the Stata statistical software (STATA, IC 18.0, STATACorp LP, Texas, TX, USA).

## 3. Results

In the period between June 2006 and December 2020, 310 patients underwent CRS+HIPEC for PM-CRC.

### 3.1. Baseline Characteristics

Among the 310 included patients, 181 (58.3%) had S-PM and 129 (41.6%) had M-PM. A larger proportion of patients with S-PM compared to patients with M-PM had mucinous adenocarcinoma (n = 31 (17.1%) vs. n = 10 (7.7%), *p* = 0.05). Furthermore, a significantly higher proportion of patients with S-PM received neoadjuvant chemotherapy (n = 103 (56.9%) vs. n = 41 (31.8%), *p* < 0.01), whereas extraperitoneal metastases prior to CRS+HIPEC were more prevalent in patients with M-PM (n = 34 (26.4%) vs. n = 19 (10.5%), *p* < 0.01).

Before CRS+HIPEC, 88.4% (n = 114/129) of M-PM patients underwent a colonic resection compared to 39.8% (n = 72/181) of S-PM patients. Notably, a larger proportion of the colonic resections in the S-PM patients (68%, n = 49/72) were right-sided, compared to the colonic resections in the M-PM group (44%, n = 51/114).

Fifty-one patients were followed at their local hospital outside the Central Denmark Region after the first postoperative control at AUH, among whom 9 patients were identified through the national pathology data with histopathological verified recurrence and 8 patients were referred for re-evaluation due to recurrence, while 34 patients were censored at the last day of follow-up in the Central Denmark Region.

Further baseline characteristics are outlined in Table 1.

### 3.2. Pattern of Recurrence

**Figure 1:**
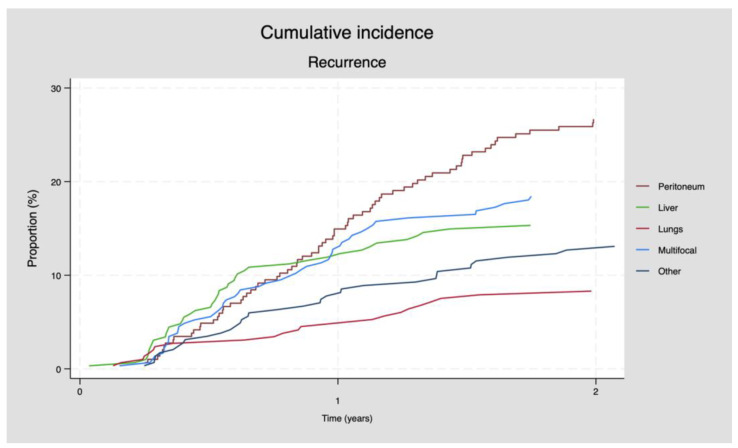
The cumulative incidence proportion of recurrence for the total population.

During a median follow-up time of 10.3 months (range: 0.3–93.1), 247 (79.7%) patients had recurrent disease detected, among whom 80 (32.4%) patients had isolated peritoneal recurrence. In total, 169 (68.4%) patients experienced extraperitoneal recurrence, among whom 56 (22.7%) patients had multifocal recurrence (see Table 2). Overall, 80% of the patients had recurrence within 24 months after CRS+HIPEC (Figure 1).

### 3.3. Disease-Free Survival

**Figure 2:**
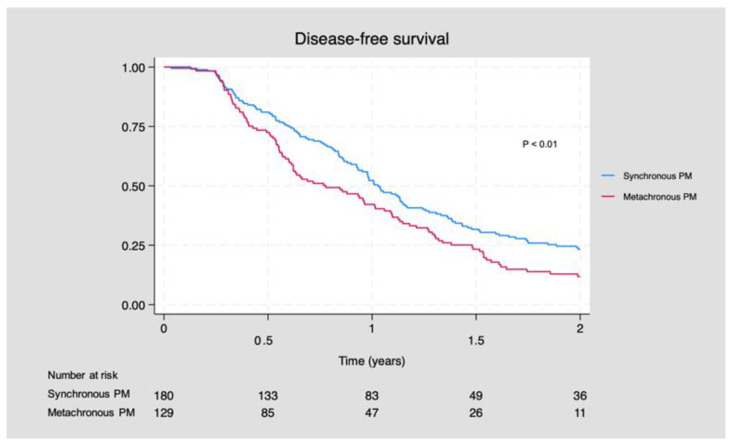
Disease-free survival for patients with synchronous and metachronous peritoneal metastases.

The median DFS for patients with S-PM was 12.4 months (95% CI: 11.1, 13.8) compared to 9.4 months (95% CI: 7.3, 12.1) for patients with M-PM (*p* < 0.01). In general, patients with M-PM demonstrated a reduced DFS compared to patients with S-PM (*p* < 0.01) (Figure 2) (Supplemental, Table S1).

### 3.4. Overall Survival

**Figure 3:**
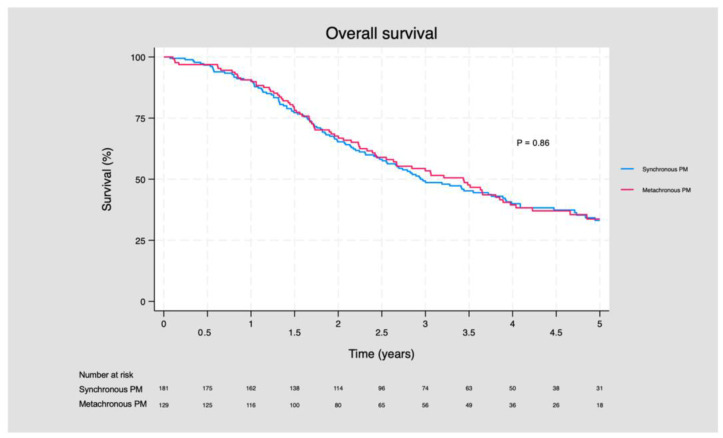
Overall survival for patients with synchronous and metachronous peritoneal metastasis.

The median OS for patients with S-PM was 38.4 months (95% CI: 31.2, 46.8), and it was 40.8 months (95% CI: 28.8, 46.8) for patients with M-PM (*p* = 0.58). In general, the OS did not differ between patients with S-PM compared to patients with M-PM (*p* = 0.86) (Figure 3).

There was no significant difference between the 3- and 5-year OS. The three-year OS for patients with S-PM was 49.3 months (95% CI: 41.6, 56.6), and it was 53.4 (95% CI: 44.1, 61.9) months for patients with M-PM (*p* = 0.80). The five-year OS for patients with S-PM was 33.2 months (95% CI: 25.5, 41.0), and it was 33.7 months for patients with M-PM (*p* = 0.50) (Supplementary Material, Table S2).

### 3.5. Subanalyses

Excluding patients with extraperitoneal metastatic disease prior to CRS+HIPEC, the DFS was still lower in patients with M-PM (*p* = 0.05), and the OS times were similar between patients with S-PM and M-PM (*p* = 0.80).

The stratification of patients by amendment to neoadjuvant chemotherapy (yes/no), DFS (*p*= 0.76) and OS (*p* = 0.92) was similar between patients (receiving neoadjuvant treatment/no neoadjuvant treatment).

We compared the DFS and OS between patients with mucinous adenocarcinoma (n = 41) and adenocarcinoma (n = 227); patients with mucinous adenocarcinoma tended to have an impaired DFS compared to patients with adenocarcinoma; however, it was non-significant (*p*-value = 0.06). There was no difference in the OS between patients with mucinous adenocarcinoma and adenocarcinoma (*p* = 0.24).

## 4. Discussion

The current study demonstrated that the recurrence pattern did not differ between patients with S-PM and M-PM, with extraperitoneal recurrence being most frequent. Isolated peritoneal recurrence was observed among one-third of patients with recurrence. Patients with M-PM had a reduced disease-free survival compared to patients with S-PM. However, the overall survival was similar between patients with S-PM and M-PM.

The demonstrated overall recurrence rate in our study was 79.7% at a median follow-up of 10.3 months. This correlates with other studies, which demonstrate recurrence rates between 62 and 77% with a median follow-up time of 25–39 months [13,15,17]. We demonstrated that app. 68% of the patients with recurrence had extraperitoneal disease, which is higher compared to other studies, who found that 53–59% of the patients had extraperitoneal recurrence [13,15,17]. Our findings of isolated peritoneal recurrence of ~32% is in accordance with the findings in the literature that show that the occurrence of isolated peritoneal recurrence varies between 34 and 47% [13,15,17]. The high rates of extraperitoneal recurrence may reflect several issues: First, a standardized follow-up protocol including frequent contrast-enhanced CT will induce a detection of recurrence, especially the extraperitoneal type, because the detection of PM is challenging. Contrast-enhanced CT and magnetic resonance imaging (MRI) are reported with a variable sensitivity in detecting PM of gastrointestinal, gynecological and other origins [26,27]. Laghi et al. [26] showed in meta-analysis from 2017 that the pooled sensitivity of contrast-enhanced CT was reported to be 83% (95% confidence interval: 79–86%), whereas the pooled sensitivity of MRI was reported to be 86% (95% confidence interval: 78–93%). Likewise, Koumpa et al. [27] reported the sensitivity of contrast-enhanced CT to detect CRC-PM to be between 11 and 96%. Both reviews found that the sensitivity of contrast-enhanced CT to detect PM differs according to the anatomical location of the PM. Furthermore, the diagnostic accuracy was found to be impacted by imaging methodologies and variables, i.e., scanner and acquisition for protocols, MRI sequences and the experience of the investigating radiologist.

Second, it must be noted that despite multifocal recurrence being frequent, peritoneal recurrence occurs in ~32% of our study population, which underlines that PM is a locoregional disease requiring targeted treatment to the peritoneal surface, such as CRS combined with HIPEC [28,29]. Third, the high recurrence rates demonstrate that characteristics of the peritoneal cavity and histopathological and genomic characteristics of the tumor cells remain unknown yet require further research [28]. In the current study, we found that a larger proportion of patients with S-PM underwent a right-sided colonic resection at primary surgery (68 vs. 44%), yet the recurrence rate and location may also rely on characteristics in the primary tumor (e.g., tumor location and histology). In this study, a larger proportion of patients with S-PM had mucinous adenocarcinoma compared to the M-PM-group (17.1% vs. 7.7%). Mucinous adenocarcinomas are more frequent in the right colon [30,31], which is in accordance with the higher proportion of right-sided colonic resection among S-PM patients in our study. The literature has shown that mucinous adenocarcinomas are more frequently diagnosed when they are already in a more advanced stage [31] and usually have poorer responses to chemotherapy compared to non-mucinous tumors [32]. The higher proportion of mucinous adenocarcinoma in the S-PM group reflects the more advanced stage of disease at the time of diagnosis. Furthermore, mucinous adenocarcinoma has been associated with a poorer prognosis with a decreased DFS and OS compared to non-mucinous adenocarcinomas [33,34]. This could be a confounding factor. However, with the present sample size we were unable to demonstrate that mucinous adenocarcinoma is positively associated with DFS and OS.

Finally, the high recurrence rates demonstrate and underline that PM is a metastatic disease associated with poorer outcomes compared to extraperitoneal metastatic disease involving organs such as the liver [13,35]. It calls for a continuous focus and refinement of current treatments, such as CRS in combination with HIPEC.

We demonstrated a decreased median DFS for M-PM compared to S-PM (9.4 vs. 12.5 months, *p* < 0.01). A significantly lower DFS for M-PM compared to S-PM has also been reported by Hentzen et al. [15], who found it was 11 vs. 15 months for M-PM vs. S-PM. The inferior DFS among patients with M-PM may be due to several factors: first, it may reflect that patients with M-PM have more widespread disease, for example, hematogenous microspread at the time of debut. Second, all patients with M-PM had undergone previous surgery due to the primary tumor, with the potential consequence of intraabdominal adherence and entrapment. This would challenge the cytoreductive surgery, due to the inaccessibility of the intra-abdominal cavity leading to potential persisting tumor nodules impairing the completeness of the cytoreduction. Furthermore, previous surgery could increase the potential intra- and postoperative complication rates and thereby impact the long-term survival. However, the OS was not impaired in patients with M-PM compared to patients with S-PM.

In contrast to our findings, Dietz et al. [14] as well as Hassan et al. [17] found no significant difference in the DFS between S-PM and M-PM (9 months in S-PM vs. 8 months in M-PM and 11 months in S-PM vs. 12 months in M-PM, respectively). The inconsistent results regarding differences in DFS between S-PM and M-PM have been suggested to reflect the different chemotherapeutic regimes [14]. To date, there is no randomized controlled trial investigating the effect of adjuvant systemic chemotherapy on patients undergoing CRS+HIPEC. Hypothetically, adjuvant chemotherapy is potentially beneficial to eradicate systemic micrometastases and post-surgical residual cancer cells, yet due to disadvantages such as systemic toxicity, increased postoperative morbidity, a decreased quality of life and higher costs, the question of adjuvant chemotherapy as a standard treatment remains unanswered [36]. We found that a significantly larger proportion of patients with S-PM received preoperative chemotherapy, and it might have an impact on the DFS. However, our subanalysis demonstrated no difference in the DFS and OS when stratifying for neoadjuvant chemotherapy. The results of the phase 3 trial of CAIRO VI study [37] are awaited to conclude more on the role of preoperative chemotherapy.

The inconsistent results regarding differences in the DFS between S-PM and M-PM may also be caused by differences in the treatment throughout the years and thus differences in the program for follow-up. In the study period, CRS+HIPEC as a treatment modality has evolved, including the surgical expertise and refinement, the oncological treatment modalities and the treatment indications including patients with extraperitoneal disease. In our study, all patients were operated on by the same group of surgeons, and patients routinely underwent CT 3, 6, 12, 18 and 24 months and 3, 4 and 5 years after the surgery. Hentzen et al. [15] followed the patients with measurement of carcinoembryonic antigen (CEA), and CT was only performed in case recurrence was suspected (e.g., clinical symptoms and increasing CEA levels). Currently, a follow-up program based on circulating tumor DNA is being investigated, yet this follow-up method has not been proven reliable to detect peritoneal metastases [38]. Thus, no optimal follow-up modality to detect peritoneal metastases early is available.

In comparison, other studies, such as Braam et al. [16], have reported recurrent disease in 46% of the patients with a median follow-up time of 26.6 months. Direct comparison is not reasonable, since important factors such as the PCI are not reported by Braam et al. [16]. Currently, the strongest prognostic factors for survival after CRS+HIPEC are the PCI and the completeness of the performed cytoreduction, and it is determined during the surgical procedure. These factors cannot be used in the preoperative setting to assess which patients will benefit from CRS+HIPEC.

Despite potential differences in the DFS, we found a similar OS between S-PM and M-PM (38 vs. 41 months). We demonstrated a higher OS than Hentzen et al. [15], but they also found no difference in OS between patients with S-PM and M-PM (34 months vs. 33 months). In contrast, Dietz et al. [14] found that S-PM had a poorer prognosis with an inferior OS compared to M-PM. This is in accordance with the findings by Hassan et al. [17], who found that patients with M-PM had a significantly higher median OS of 51 month versus 35 months for patients with S-PM [17].

It has been suggested that the poor prognosis may be due to the aggressive presentation of S-PM. The role of preoperative systemic chemotherapy for patients with S-PM remains unsure. Zhou et al. showed [39] in a small study with 52 patients that patients who received chemotherapy prior to CRS+HIPEC had a higher 2-year OS rate than those who underwent CRS+HIPEC without preoperative chemotherapy (67.4% vs. 32.2%). Furthermore, the patients receiving systemic chemotherapy were more likely to achieve complete cytoreduction (80.0% vs. 46.9%) [39]. The results should be interpreted with caution due to the low number of patients included in the study. Furthermore, due to the design of the study by Zhou et al. [39], there is no information about the patients who received neoadjuvant chemotherapy prior to CRS+HIPEC but had disease progression, which may translate into a potential selection bias leading to the patients with the most favorable prognoses being included in the study.

In the French RCT, PRODIGE 7 [10], 83% of the patients received neoadjuvant systemic chemotherapy before CRS+HIPEC. In total, 89% of the patient undergoing CRS+HIPEC achieved complete macroscopic cytoreduction. They showed a median OS of 41.7 months and a 5-year survival rate of 39.4%. The PRODIGE-7 trial [10] showed a slightly better median OS and 5-year survival rate than we did. In our study, only 45% of the patients received neoadjuvant systemic chemotherapy, but 98.4% had complete cytoreduction at CRS+HIPEC. One explanation could be that patients with disease progression during neoadjuvant chemotherapy will not be offered CRS+HIPEC; thus, the patient cohort who will be offered CRS+HIPEC will be a selective cohort with a more favorable prognosis.

PM is considered a heterogenous disease, which is reflected in the treatment challenge. In the current study, we demonstrated several heterogenous characteristics between patients with S-PM and M-PM. Despite the differences between the two groups, patients with S-PM and M-PM are offered the same treatment with CRS+HIPEC and chemotherapy in a pre- or postoperative setting. In the literature and internationally, S-PM and M-PM are considered the same disease entity. We found no differences in the OS for patients with S-PM and M-PM, making comparison of the two groups reasonable.

### Strengths and Limitations

This study is mainly based on a prospective database and included a relatively large sample. To our knowledge, we are the first to study the difference in the recurrence patterns of S-PM and M-PM in CRC patients undergoing CRS+HIPEC in such a large cohort. All the patients, regardless of if they had S-PM or M-PM, had the same follow-up program. In the last 10 years, the CRS+HIPEC procedure was carried out by few experienced surgeons, which increased the chances of complete cytoreduction. The cohort includes patients from the very early beginning of CRS+HIPEC as a treatment modality, and, expectedly, the surgical technique has improved through the years as the experience with the procedure has improved.

For patients operated on in the very early beginning of CRS+HIPEC, we had no information about the PCI score, and therefore we cannot adjust the results for this factor. Most of the patients with recurrence were re-evaluated at MDT. If a patient’s follow-up was scheduled at a local hospital outside the Central Denmark Region, we had no access the patient’s health record due to the different health record systems in Denmark. The only information available was the vital status and access to the national pathology data. Therefore, some patients with recurrence but without histological confirmation could potentially be missed. We have taken that into account by censoring the patients from the DFS analyses.

Unfortunately, we did not have information about mutation status of KRAS/NRAS, BRAF, MSI and HER2 for the entire cohort, which could have contributed to improve the understanding of the recurrence pattern of PM after CRS+HIPEC.

## 5. Conclusions

Long-term survival was achievable in CRC patients with PM after CRS+HIPEC. We found no difference in recurrence patterns between S-PM and M-PM, but, overall, the majority of patients with recurrent disease had extraperitoneal recurrence (67%). Though the DFS was inferior for patients with M-PM, the OS did not differ between patients with S-PM and M-PM.

## Figures and Tables

**Table 1:** Baseline characteristics of CRC patients with peritoneal metastases undergoing CRS+HIPEC.

Variable	Patients with Synchronous PM n = 181	Patients with Metachronous PM n = 129	*p*-Value	Total n = 310
**Sex (n, %)** Male Female	83 (45.9) 98 (54.1)	57 (44.2) 72 (55.8)	0.77	140 (45.2) 170 (54.8)
**Age (median, IQR)**	62 (17)	64 (15)	0.35	63 (16)
**ASA score (n, %)** 1 2 3	68 (37.6) 103 (56.9) 10 (5.5)	35 (27.1) 88 (68.2) 6 (4.7)	0.13	103 (33.2) 191 (61.6) 16 (5.2)
**Previous cancer-related surgery (n, %)** None Colonic resection • Right-sided • Left-sided • Colectomy Rectal resection Laparoscopy only ^1^ Laparotomy only Alleviating surgery ^2^ Gynaecologic surgery N/A	44 (24.3) 72 (39.8) 49 (68.1) 20 (27.8) 3 (4.2) 7 (3.9) 19 (10.5) 5 (2.8) 31 (17.1) 3 (1.6) 0 (0.0)	0 (0.0) 114 (88.4) 51 (44.7) 60 (52.6) 3 (2.6) 14 (10.9) 0 (0.0) 0 (0.0) 0 (0.0) 0 (0.0) 1 (0.7)	<0.01	44 (14.2) 186 (60.0) 100 (53.8) 80 (43.0) 6 (3.2) 21 (6.8) 19 (6.1) 5 (1.6) 31 (10.0) 3 (1.0) 1 (0.3)
**Histology of CRS (n, %)** Adenocarcinoma, CRC Mucinous adenocarcinoma No malignancy ^3^	122 (67.4) 31 (17.1) 28 (15.5)	105 (81.4) 10 (7.7) 14 (10.9)	0.02	227 (71.6) 41 (12.9) 42 (13.6)
**Extraperitoneal metastases prior to CRS+HIPEC (n, %)** No ^4^ Yes	162 (89.5) 19 (10.5)	95 (73.6) 34 (26.4)	<0.01	257 (82.9) 53 (17.1)
**Preoperative chemotherapy (n, %)** No Yes	78 (43.1) 103 (56.9)	88 (68.2) 41 (31.8)	<0.01	166 (53.6) 144 (45.4)
**Year of CRS+HIPEC (n, %)** 2006–2010 2011–2015 2016–2020	12 (6.6) 42 (23.2) 127 (70.2)	5 (3.9) 26 (20.1) 98 (76.0)	0.32	17 (5.5) 68 (21.9) 225 (75.6)
**Median time from resection of primary tumor to CRS+HIPEC** (months, IQR)	4.2 (4.8) ^5^ n = 159	19.5 (16.1) ^5^ n = 127	<0.01	6.2 (13.4) ^5^ n = 286
**PCI score** <6 6–10 >10 Missing ^6^	58 (32.0) 42 (23.2) 32 (17.7) 49 (27.1)	40 (31.0) 40 (31.0) 19 (14.7) 30 (23.3)	0.46	98 (31.6) 82 (26.5) 51 (16.4) 79 (25.5)
**Hyperthermic chemotherapy** Mitomycin Oxaliplatin None	88 (48.6) 92 (50.8) 1 (0.6)	74 (57.4) 55 (42.6) 0 (0.0)	0.24	162 (52.3) 147 (47.4) 1 (0.3)
**Completeness of cancer resection (CCR)** CC0	178 (98.3)	127 (98.5)	0.94	305 (98.4)

^1^ Including polypectomy (n = 1) or appendectomy (n = 3). ^2^ Alleviating surgery: stent (n = 10), enteroenterostomy (n = 6), stoma (n = 17). ^3^ Including no malignancy and other pathology (n = 1). Neo-adjuvant chemotherapy was administered to 73.8% (n = 31/42) of patients. PM or ovarian metastases were histologically confirmed at laparoscopy or previous surgery (n = 11). ^4^ Before 2016, patients with extraperitoneal metastatic disease were not offered CRS+HIPEC. ^5^ For a few patients, the date of primary surgery was not available. ^6^ The PCI score was registered from 2015.

**Table 2:** The pattern of recurrence among patients with synchronous and metachronous peritoneal metastases (PM).

Pattern of Recurrence	Synchronous PM n = 181	Metachronous PM n = 129	*p*-Value	Total n = 310
**Median follow-up** (months, IQR)	11.7 (14.2)	7.6 (11.2)	-	10.3 (12.9)
**Recurrence, n (%)**				
No Yes	40 (22.1) 141 (77.9)	23 (17.8) 106 (82.2)	0.68 0.41	63 (20.3) 247 (79.7)
**Diagnostic modality, n (%)**				
Clinical Computer tomography Perioperatively Lab incl. pathology	5 (3.5) 119 (84.4) 10 (7.1) 7 (5.0)	6 (5.7) 95 (89.6) 2 (1.9) 3 (2.8)	-	11 (4.4) 214 (86.6) 12 (4.9) 10 (4.0)
**Location of recurrence, n (%)**				
Isolated peritoneal Peritoneal and extraperitoneal Isolated extraperitoneal • Liver • Lungs • Other ^1^	50 (35.5) 26 (18.4) 65 (46.1) 26 (40.0) 16 (24.6) 23 (35.4)	30 (28.3) 30 (28.3) 46 (43.4) 18 (39.1) 10 (21.7) 18 (39.1)	0.51 0.38 0.78 - - -	80 (32.4) 56 (22.7) 111 (44.9) 44 (39.6) 26 (23.4) 41 (36.9)

^1^ Abdominal wall, uterus, spleen, bones, cerebrum and adrenal gland.

## Data Availability

Data are contained within the article.

## Supplementary Materials

The following supporting information can be downloaded at https://www.mdpi.com/article/10.3390/cancers16030631/s1, Table S1: Disease-free survival for patients with S-PM and M-PM. Table S2: 1-, 3- and 5-year overall survival for patients with S-PM and M-PM.
