# Supplementary material for: Recurrence and Survival Following Cytoreductive Surgery and Hyperthermic Intraperitoneal Chemotherapy for Synchronous and Metachronous Peritoneal Metastases of Colorectal Origin
Source: Cancers (Basel). 2024 Feb 1;16(3):631. doi: 10.3390/cancers16030631 (PMC10854638; doi:10.3390/cancers16030631)
Supplement: Supplementary file 1 [file cancers-16-00631-s001.zip › Supplemental materials. Table S1 og S2 - Disease-free survival - Overall survival - 30-12-2023.pdf]

TABLE S1. Disease-free survival for patients with synchronous and metachronous peritoneal metastases.

Time (months)	Synchronous PM n=181	Metachronous PM n=129	P-value	Total n=310
0-6	81.0 (74.2; 86.2)	72.5 (63.6; 79.6)	0.9	77.5 (72.2; 81.9)
6-12	52.3 (44.4; 59.6)	42.2 (33.1; 50.9)	0.01	48.1 (42.1; 53.8)
12-18	31.7 (24.6; 39.0)	23.3 (16.0; 31.4)	0.8	28.2 (23.0; 33,6)
18-24	23.3 (17.0; 30.1)	11.8 (6.6; 18.7)	0.02	18.6 (14.2; 23.5)

TABLE S2. Overall survival for patients with synchronous and metachronous peritoneal metastases.

Time (year)	Synchronous PM n= 187	Metachronous PM n=130	P-value	Total n=317
**1**	90.4 (84.7; 93.6)	90.7 (84.2; 94.6)	0.9	90.3 (86.4; 93.1)
**3**	49.3 (41.6; 56.6)	53.4 (44.1; 61.9)	0.8	51.0 (45.1; 56,6)
**5**	33.2 (25.5; 41.0)	33.7 (24.5; 43.2)	0.5	33.3 (27.4; 39.4)
